# Inhibition of Acute mGluR5-Dependent Depression in Hippocampal CA1 by High-Frequency Magnetic Stimulation

**DOI:** 10.3390/brainsci14060603

**Published:** 2024-06-14

**Authors:** Norman Holl, Marco Heerdegen, Volker Zschorlich, Rüdiger Köhling, Timo Kirschstein

**Affiliations:** 1 Oscar Langendorff Institute of Physiology, University Medicine Rostock, University of Rostock, Gertrudenstrasse 9, 18057 Rostock, Germany; norman.holl@med.uni-rostock.de (N.H.); marco.heerdegen@uni-rostock.de (M.H.); ruediger.koehling@uni-rostock.de (R.K.); 2 Institute of Sport Sciences, University of Rostock, Am Waldessaum 23a, 18057 Rostock, Germany; volker.zschorlich@uni-rostock.de; 3 Institute of Sport Sciences, Carl von Ossietzky University of Oldenburg, Ammerländer Heerstraße 114-118, 26129 Oldenburg, Germany

**Keywords:** CHPG, DHPG, NMDA, group I mGluR, adolescent rat, gabazine

## Abstract

High-frequency magnetic stimulation (HFMS) applied directly to the hippocampal slice preparation in vitro induces activity-dependent synaptic plasticity and metaplasticity. In addition, changes in synaptic transmission following HFMS involve the activation of N-methyl-D-aspartate and metabotropic glutamate receptors (mGluR). Here, we asked whether a short period of HFMS (5 × 10 delta-burst trains, duration of ~1 min) could alter mGluR5-mediated depression at Schaffer collateral–CA1 synapses in the acute brain slice preparation at 30 min after HFMS. To this end, we obtained field excitatory postsynaptic potential (fEPSP) slopes from Schaffer collateral–CA1 synapses after HFMS or control. First, we demonstrated that activity-dependent plasticity following HFMS depends on the slice orientation towards the magnetic coil indicating specific ion fluxes induced by magnetic fields. Second, we found that the mGluR5-specific agonist (RS)-2-chloro-5-hydroxyphenylglycine reduced the field excitatory postsynaptic potential (fEPSP) slopes in control slices but rather enhanced them in HFMS-treated slices. In contrast, the compound (S)-3,5-dihydroxyphenylglycine acting at both mGluR1 and mGluR5 reduced fEPSP slopes in both control and HFMS-treated slices. Importantly, the mGluR-dependent effects were independent from the slice-to-coil orientation indicating that asynchronous glutamate release could play a role. We conclude that a short period of HFMS inhibits subsequently evoked mGluR5-dependent depression at Schaffer collateral–CA1 synapses. This could be relevant for repetitive transcranial magnetic stimulation in psychiatric disorders such as major depression.

## 1. Introduction

Activity-dependent synaptic plasticity is the major molecular hallmark of information storage in the central nervous system and is classically induced by medium-to-high frequency electrical stimulation applied to afferent fibers, therefore often referred to as electrically induced plasticity [1,2]. While N-methyl-D-aspartate receptor (NMDAR) activation was initially thought to be instrumental for its induction, alternative routes of establishing synaptic plasticity such as metabotropic glutamate receptor (mGluR)-dependent mechanisms and bath application of group I mGluR agonists [3,4,5] were discovered. Therefore, this latter form is often referred to as chemically induced plasticity and shares only some of the mechanisms with those involved in electrically induced plasticity. Hence, both forms are regarded as largely independent from one another and sometimes co-exist at the same synapse such as the Schaffer collateral–CA1 synapse [6]. 

A large body of evidence indicates that repetitive magnetic stimulation induces synaptic potentiation both in vitro [7,8,9,10,11,12] and in vivo [13,14]. Earlier, this laboratory could demonstrate that high-frequency magnetic stimulation (HFMS) induced potentiation at Schaffer collateral–CA1 synapses that was postsynaptic in nature (it was prevented by the NMDAR blocker D-AP5, and presynaptic fiber volleys remained unchanged) [8]. Beyond that, HFMS by itself leading to potentiation occluded subsequent potentiation by electrical stimulation [10]. As expected, this occlusive priming effect of HFMS was NMDAR-dependent. Intriguingly, however, it also involved mGluR-dependent pathways given that electrically induced potentiation after magnetic priming almost resumed in the presence of broad-spectrum mGluR inhibition [10]. By implication, these findings suggest that HFMS may not only affect synaptic plasticity but involve mGluR activation as well. While it is apparent that medically used drugs may interfere with mGluR-dependent plasticity, there is little awareness of such an interaction between physical stimulation and chemically induced plasticity. Given that repetitive transcranial magnetic stimulation is approved for major depression disorder in the United States as well as in many European countries, and that increased mGluR5 is involved in depressive animal models [15], such an interaction could have clinical relevance in psychiatric diseases. 

To examine the interplay between magnetic stimulation and mGluR activation directly, we tested the effect of HFMS on the subsequent response of the mGluR agonist to bath application. We hypothesized that asynchronous glutamate release during HFMS could be responsible for mGluR activation. Therefore, firstly, we asked whether the orientation of the Schaffer collateral pathway had an impact on the magnitude of this effect. We found significantly different potentiation between two mirror-image slice positions favoring the hypothesis of a slice-to-coil orientation effect. Secondly, we administered group I mGluR agonists and found significant interference between magnetic stimulation and mGluR-depression only for the mGluR5 agonist CHPG, but not for the mGluR1/5 agonist DHPG, and—importantly—this was independent of the slice-to-coil orientation. 

## 2. Materials and Methods

### 2.1. Slice Preparation and Maintenance

To obtain horizontal hippocampal slices, 2–3-month-old male Wistar rats (Charles-River, Sulzfeld, Germany) were anesthetized with diethylether and decapitated. The brain was rapidly removed and brain slices (400 µm) were cut using a vibratome (Campden Instruments, Loughborough, UK) in an almost 0 °C cold dissection solution composed of (in mM) 87 NaCl, 25 NaHCO_3_, 2.5 KCl, 1.25 NaH_2_PO_4_, 0.5 CaCl_2_, 7 MgCl_2_, 10 glucose and 75 sucrose (pH = 7.4, 308–314 mosmol/kg H_2_O). The hippocampus proper was then microdissected from the brain slice and allowed to equilibrate in the dissection solution at room temperature for at least 60 min. 

Following the equilibration at room temperature, slices were transferred to a Haas-type interface chamber (BSC-HAT, Harvard Apparatus, Holliston, MA, USA) perfused with artificial cerebrospinal fluid (ACSF) consisting of (in mM) 124.5 NaCl, 26 NaHCO_3_, 3 KCl, 1.25 NaH_2_PO_4_, 2.5 CaCl_2_, 1.3 MgCl_2_ and 10 glucose (pH = 7.4, 308–314 mosmol/kg H_2_O). Unless otherwise indicated, all chemicals were obtained from Sigma-Aldrich (Taufkirchen, Germany). The GABA_A_ receptor antagonist gabazine (0.5 µM, Tocris, Bristol, UK) was added to the ACSF to minimize GABAergic contamination and thereby facilitating mGluR-dependent depression [3]. The temperature was controlled (TC-10, npi electronic GmbH, Tamm, Germany) and held at 32 ± 1 °C. The perfusion rate was adjusted to 2 mL/min (MCM-500, MC-Medizintechnik, Alzenau, Germany). 

### 2.2. Magnetic Stimulation

**Figure 1:**
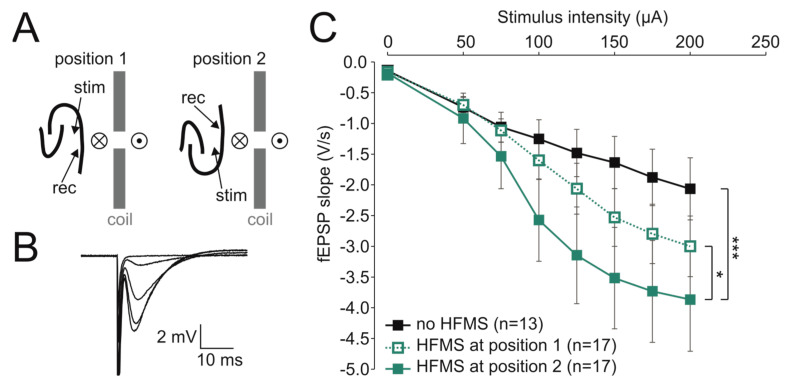
High-frequency magnetic stimulation (HFMS) potentiated synaptic transmission in a slice-to-coil orientation-specific manner. (**A**) Two mirror-image positions of hippocampal slices with respect to the magnetic coil. ⊗ indicates magnetic field lines pointing into the page (i.e., north pole). ⊙ indicates magnetic field lines emerging from the page (i.e., south pole). Position 1 was established in [8]. (**B**) Representative traces showing fEPSPs for 0, 50, 100, 150 and 200 µA after HFMS at position 1. (**C**) Input–output relationship of Schaffer collateral–CA1 synapses under control conditions (no HFMS) or following HFMS (either at position 1 or at position 2). The effect of HFMS at position 2 (*** *p* < 0.001), but not at position 1 (*p* = 0.195), was statistically significant. Moreover, there was a statistically significant difference between both slice-to-coil orientations (* *p* = 0.015; two-way ANOVA with Tukey’s post hoc test).

A subset of slices was subjected to magnetic stimulation prior to establishing the extracellular recording. After at least 30 min equilibration in the Haas-type interface chamber perfused with ACSF and 0.5 µM gabazine, a circular magnetic coil (MC-125, diameter 125 mm, thickness 11.3 mm, MagVenture, Skovlunde, Denmark) was placed perpendicular to the slice and parallel to the CA1 pyramidal layer at a distance to the slice of 9–10 mm [8]. Hence, the magnetic field lines perforated the slice from above and were oriented orthogonally to the pathway of the Schaffer collaterals. While the position of the coil was invariably oriented as shown in Figure 1A [8], we randomly inserted the slice either in the same way as in our previous study (position 1 in Figure 1A) or in a mirror-inverted one (position 2 in Figure 1A). In contrast to our previous study [8], we first applied magnetic stimulation, removed the coil and then established the electrophysiological recording (see Section 2.3) to rule out indirect effects via electrodes in place. 

Magnetic stimulation was performed as in our earlier study [8] and consisted of 5 repetitions (10 s apart) of 10 trains (1 s apart), each of which had 20 pulses at 100 Hz (i.e., train duration of 200 ms). The intensity of the magnetic stimulator (MagPro R100, MagVenture) was set to 50%, which was equivalent to the output of 60–75 A/µs [8]. This stimulator typically yields a magnetic flux density of 4100 mT. Since the capacitor can maximally be loaded with 300 J, we assume that we delivered 150 J per impulse. During magnetic stimulation the coil temperature increases and impulse delivery automatically stops at 40 °C coil temperature. To prevent an interruption of magnetic stimulation before completing the protocol, we pre-cooled the coil in a 16 °C environment prior to magnetic stimulation. Following the magnetic stimulation, the coil was removed to allow establishing the electrophysiological recordings. 

### 2.3. Extracellular Recording and mGluR-Depression

After having removed the magnetic coil, electrodes for electrical stimulation and for recording of Schaffer collateral–CA1 synapses were set on the slice. Both electrodes consisted of borosilicate glass capillaries pulled to reach a tip resistance of 2–3 MΩ (PIP5, HEKA Elektronik, Lambrecht, Germany) and were filled with ACSF and Ag/AgCl wires. The recording electrode was connected to the amplifier (EXT-10-2F, npi) and the stimulation electrode was connected to the stimulus isolator (ISO-STIM 01D, npi). Both stimulation and recording electrodes were placed into the CA1 stratum radiatum to stimulate Schaffer collaterals and to record from CA1 dendrites. Excitatory postsynaptic field potentials (fEPSP) evoked by electrical afferent stimulation were lowpass-filtered at 1 kHz, digitized and analyzed (Micro1401 and Signal 2.16 software, CED, Cambridge, UK). 

Each recording started with an input–output relationship using increasing stimulus intensities (from 50 to 200 µA in 25 µA steps). Then, the stimulus intensity was adjusted to yield an fEPSP amplitude of 40–50% of the maximum amplitude obtained in this input–output experiment. This was called baseline stimulus intensity and retained until a 30 min period had elapsed since the HFMS. Then, the mGluR5 agonist (RS)-2-chloro-5-hydroxyphenylglycine (CHPG, 150 µM, Tocris) or the mGluR1/5 agonist (S)-3,5-dihydroxyphenylglycine (DHPG, 100 µM, Tocris) were applied for 15 min to obtain the mGluR effect on synaptic transmission [3,4]. Stimulation was performed every 30 s and all fEPSP traces were processed to analyze the maximum initial negative slope which was normalized to the baseline value of this slice. The mGluR effect was assessed as the mean relative fEPSP slope during the last 3 min (i.e., 12–15 min after the beginning of mGluR agonist application, average of 7 sweeps). 

### 2.4. Statistics

Statistical analyses were performed using JMP 14 (SAS Institute Inc., Cary, NC, USA). All data are expressed as the mean ± SEM. To test for normal distribution, the Shapiro–Wilk test was used. Since all data were normally distributed, differences were analyzed using Student’s *t*-test or ANOVA with Tukey’s post hoc test. The level of statistical significance was set to 0.05.

## 3. Results

First, we aimed to confirm that high-frequency magnetic stimulation (HFMS) leads to synaptic potentiation. The input–output relationship from HFMS-treated slices differed significantly from untreated control slices (F = 9.721, *p* < 0.001, two-way ANOVA with Tukey’s post hoc test). Since we were interested in whether the slice orientation affected synaptic potentiation, we compared the experiments using a slice position as previously used (position 1 in Figure 1A [8], sample trace in Figure 1B) with the mirror-inverted position (position 2 in Figure 1A). This comparison revealed that the slice orientation was critical as the input–output relationship at position 1 did not statistically differ from that under control conditions (*p* = 0.195, two-way ANOVA with Tukey’s post hoc test; Figure 1C) given the significant difference between the input–output relationships at positions 1 and 2 (*p* = 0.015; Figure 1C). 

**Figure 2:**
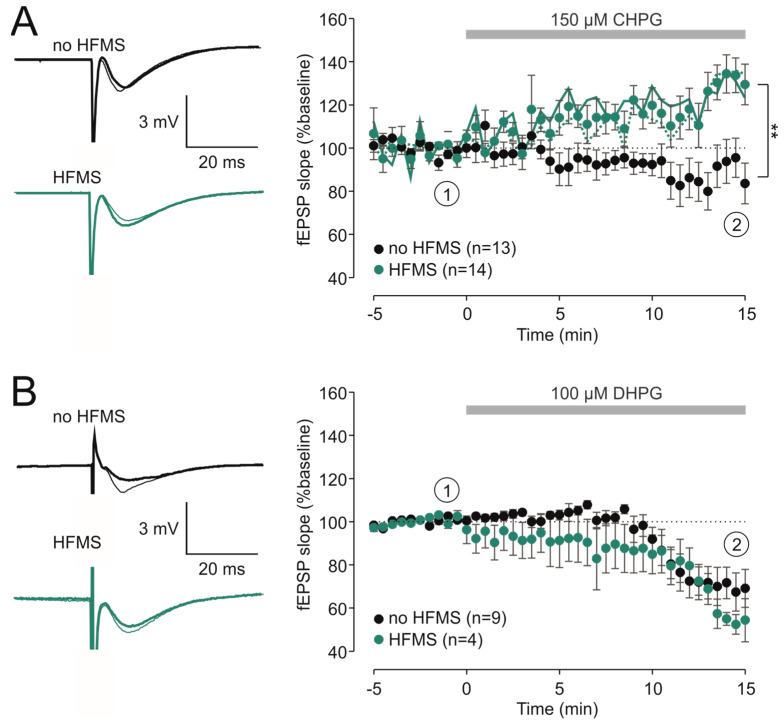
HFMS inhibits CHPG-induced depression but not DHPG-induced depression. (**A**) Right, time course of the relative fEPSP slopes (in % of baseline values) following 150 µM CHPG in slices under control conditions (no HFMS, black circles) or after HFMS (green circles). The solid and broken lines indicate the mean values for fEPSP slopes at position 1 and position 2, respectively. At the end of the experiment, there was a significant difference between control and HFMS-treated slices (t = −3.420, ** *p* = 0.002, unpaired *t*-test). Left, representative traces were taken at timepoint ① (directly before drug application, thin trace) and ② (end of application, thick trace). (**B**) Right, time course of the relative fEPSP slopes (in % of baseline values) following 100 µM DHPG in control slices (no HFMS, black circles) and HFMS-treated slices (green circles). Left, representative traces were taken at timepoint ① (directly before drug application, thin trace) and ② (end of application, thick trace).

We then set out to test the effect of the mGluR5 agonist CHPG on HFMS-treated and on control slices. To test for slice orientation, we randomly used sham stimulation, stimulation at position 1 or stimulation at position 2. In these experiments, we found that CHPG reduced the fEPSP slope in control slices (88 ± 8% of baseline, n = 13) but enhanced them in HFMS-treated slices, and that this potentiation was independent of slice orientation (position 1: 127 ± 9%, n = 8, t = −3.080, *p* = 0.006 versus control, unpaired *t*-test; position 2: 125 ± 13%, n = 6, t = −2.466, *p* = 0.025 versus control, unpaired *t*-test). Pooled data from all HFMS-treated slices as well as separate datasets for HFMS at positions 1 and 2 are depicted in Figure 2A. Hence, although the slice orientation played a significant role with respect to HFMS-induced potentiation, it did not matter for the effect of the mGluR5 agonist CHPG on Schaffer collateral–CA1 synaptic transmission. Next, we tested the effect of the mGluR1/5 agonist DHPG. As expected from the literature [3], DHPG led to robust acute depression at the end of its administration in control slices (71 ± 8%, n = 9). In contrast to CHPG, DHPG also depressed synaptic transmission in HFMS-treated slices, which was not statistically different from control (63 ± 6%, n = 4, t = 0.572, *p* = 0.579 versus control, unpaired *t*-test; Figure 2B). Again, this depression was similar in both slice positions (position 1: 67 ± 7%, n = 2; position 2: 59 ± 9%, n = 2). These data demonstrate that mGluR5-dependent depression by CHPG was inhibited by prior HFMS, but mGluR1/5-dependent depression by DHPG was intact following HFMS. 

## 4. Discussion

The present study was performed to study the effect of group I mGluR activation on Schaffer collateral–CA1 synapses following high frequency magnetic stimulation (HFMS). Firstly, we confirmed our previous results that HFMS led to synaptic potentiation and found that this potentiation was subject to the slice-to-coil orientation, albeit the potentiation using position 1 was less pronounced than previously found [8] and thus no longer significant. One needs to consider the different experimental approaches. Here, we established the recording after having applied magnetic stimulation to ensure a strictly parallel time course for all slices with or without mGluR agents. This approach made us only use the unpaired testing condition with a difference probably large enough to reach statistical significance. Secondly, we demonstrated that HFMS-treated slices exhibited impaired CHPG-induced depression but that DHPG-induced depression remained intact. In contrast to electrical plasticity, the plasticity induced by mGluR application was present regardless of the slice position.

Brief bath-applied group I mGluR agonists such as CHPG or DHPG lead to acute depression of synaptic transmission at the Schaffer collateral–CA1 synapse [3,4,16]. When directly comparing CHPG and DHPG effects on synaptic transmission, we found depression in control slices to be more pronounced with DHPG than with CHPG, reducing the fEPSP slope by 29% and 12%, respectively. By first inspection, this is inferior to data published for juvenile rats [4,17] but consistent with data from adult animals [3], confirming an age dependence. Our findings also suggest that mGluR1-mediated and mGluR5-mediated synaptic depression were complementary to each other rather than compensatory [18], probably due to an age-dependent contribution of mGluR5. There is at least some evidence for this concept: While the mGluR5-specific antagonist MPEP had no effect on DHPG-induced acute depression in adolescent rats [18], it reduced the acute depression by DHPG in adult animals [19]. Correlating with this, CHPG and DHPG both reduced the fEPSP slope to roughly 55% in juvenile rats [4], but in adult rats—even in Mg^2+^-free conditions—CHPG-induced depression yielded 50% and DHPG-induced depression 75% [3]. Alternatively, looking at differential effects of CHPG and DHPG on transmission could also point to different affinities of both compounds towards mGluR5. The in vivo potency of DHPG was six times higher than that of CHPG [20]. In addition, previous studies in adult [3] and juvenile rats [4] found comparable DHPG and CHPG effects when using 100 µM DHPG and 1 mM CHPG. Therefore, we believe that the differential effects of CHPG and DHPG in control slices could have been age-related, though we cannot exclude a more pronounced CHPG effect in control slices at a higher concentration.

Our key finding—HFMS prevents subsequent CHPG-induced depression but not DHPG-induced depression—suggests impaired mGluR5 function following magnetic stimulation. CHPG is a mGluR5-specific agonist [21], while DHPG activates both mGluR1 and mGluR5 [22,23]. Since both CHPG and DHPG led to acute depression of synaptic transmission at Schaffer collateral–CA1 synapses [3,4,16] and both mGluR1 and mGluR5 were involved [18], the most parsimonious explanation for the missing CHPG effect in HFMS-treated slices implies an impaired mGluR5 function after prior HFMS. Moreover, if each of both mGluRs had compensated for the other as previously suggested [18], this would have implied that mGluR1 alone accounted for the full effect of depression and—in turn—explained why DHPG-induced depression did not differ between control and HFMS-treated slices. One should bear in mind that there is some ambiguity as to whether DHPG-induced depression may involve receptors other than mGluR1 and mGluR5 [18,24]. Taken together, our data cannot directly prove that DHPG-induced depression in HFMS-treated slices involved only mGluR1, but the loss of the CHPG effect in HFMS-treated slices strongly suggests a compromised mGluR5 function in this tissue.

How could the HFMS-induced mGluR5 dysfunction occur? Data from studies on repetitive transcranial magnetic stimulation showed reduced expression of several genes [14] including mGluR1 in the cerebellar cortex [25] and mGluR5 in the anterior insula [26]. All these changes, however, were on the transcriptional level and thus cannot play a role in the present study. Here, the effect of HFMS was detectable after 30 min which rather indicates post-translational modifications such as phosphorylation processes. Evidence for this comes from the nucleus accumbens, where NMDAR activation enhanced Ca^2+^/calmodulin kinase II-mediated Homer 1b/c phosphorylation and thus impaired the mGluR5–Homer 1b/c interaction [27]. Having previously demonstrated NMDAR-dependent HFMS-induced plasticity [8] leads us to assume that HFMS results in asynchronous glutamate release enough to activate NMDARs and/or mGluRs. In addition, NMDAR activation itself could subsequently modify mGluR5 function. Yet, this scenario is unlikely in our study, because HFMS-induced plasticity was slice orientation-sensitive and mGluR-dependent depression was not. One possibility to address this would include experiments with NMDAR inhibition during HFMS before mGluR agonist application. This is a limitation of the present study. In addition, interpretation is also limited as paramagnetic effects of magnetic stimulation on neural circuits at the molecular level are still unknown.

## 5. Conclusions

In summary, our study adds a new mechanism involving mGluRs to the already described effects of HFMS on NMDAR-dependent glutamatergic transmission in the cortex. Given the effect of HFMS independent of slice orientation, we conclude that future studies on repetitive magnetic stimulation both in vivo and in vitro should be aware of mGluR-dependent processes at central synapses. However, increasing our knowledge on the interplay between magnetic stimulation and mGluR-dependent transmission will be relevant when used as a treatment option in psychiatric disorders. 

## Data Availability

Research data are stored in an institutional repository and will be shared upon request to the corresponding author.

## References

[1] Bliss T.V., Collingridge G.L. (1993). A synaptic model of memory: Long-term potentiation in the hippocampus. Nature.

[2] Malenka R.C., Bear M.F. (2004). LTP and LTD: An embarrassment of riches. Neuron.

[3] Palmer M.J., Irving A.J., Seabrook G.R., Jane D.E., Collingridge G.L. (1997). The group I mGlu receptor agonist DHPG induces a novel form of LTD in the CA1 region of the hippocampus. Neuropharmacology.

[4] Fitzjohn S.M., Kingston A.E., Lodge D., Collingridge G.L. (1999). DHPG-induced LTD in area CA1 of juvenile rat hippocampus; characterisation and sensitivity to novel mGlu receptor antagonists. Neuropharmacology.

[5] Bortolotto Z.A., Fitzjohn S.M., Collingridge G.L. (1999). Roles of metabotropic glutamate receptors in LTP and LTD in the hippocampus. Curr. Opin. Neurobiol..

[6] Oliet S.H., Malenka R.C., Nicoll R.A. (1997). Two distinct forms of long-term depression coexist in CA1 hippocampal pyramidal cells. Neuron.

[7] Ahmed Z., Wieraszko A. (2006). Modulation of learning and hippocampal, neuronal plasticity by repetitive transcranial magnetic stimulation (rTMS). Bioelectromagnetics.

[8] Tokay T., Holl N., Kirschstein T., Zschorlich V., Köhling R. (2009). High-frequency magnetic stimulation induces long-term potentiation in rat hippocampal slices. Neurosci. Lett..

[9] Vlachos A., Müller-Dahlhaus F., Rosskopp J., Lenz M., Ziemann U., Deller T. (2012). Repetitive magnetic stimulation induces functional and structural plasticity of excitatory postsynapses in mouse organotypic hippocampal slice cultures. J. Neurosci..

[10] Tokay T., Kirschstein T., Rohde M., Zschorlich V., Köhling R. (2014). NMDA receptor-dependent metaplasticity by high-frequency magnetic stimulation. Neural Plast..

[11] Lenz M., Galanis C., Müller-Dahlhaus F., Opitz A., Wierenga C.J., Szabó G., Ziemann U., Deller T., Funke K., Vlachos A. (2016). Repetitive magnetic stimulation induces plasticity of inhibitory synapses. Nat. Commun..

[12] Lenz M., Platschek S., Priesemann V., Becker D., Willems L.M., Ziemann U., Deller T., Müller-Dahlhaus F., Jedlicka P., Vlachos A. (2015). Repetitive magnetic stimulation induces plasticity of excitatory postsynapses on proximal dendrites of cultured mouse CA1 pyramidal neurons. Brain Struct. Funct..

[13] Tan T., Xie J., Liu T., Chen X., Zheng X., Tong Z., Tian X. (2013). Low-frequency (1 Hz) repetitive transcranial magnetic stimulation (rTMS) reverses Abeta(1-42)-mediated memory deficits in rats. Exp. Gerontol..

[14] Weiler M., Stieger K.C., Shroff K., Klein J.P., Wood W.H., Zhang Y., Chandrasekaran P., Lehrmann E., Camandola S., Long J.M. (2023). Transcriptional changes in the rat brain induced by repetitive transcranial magnetic stimulation. Front. Hum. Neurosci..

[15] Li M.-X., Li Q., Sun X.-J., Luo C., Li Y., Wang Y.-N., Chen J., Gong C.-Z., Li Y.-J., Shi L.-P. (2019). Increased Homer1-mGluR5 mediates chronic stress-induced depressive-like behaviors and glutamatergic dysregulation via activation of PERK-eIF2α. Prog. Neuropsychopharmacol. Biol. Psychiatry.

[16] Huber K.M., Roder J.C., Bear M.F. (2001). Chemical induction of mGluR5- and protein synthesis--dependent long-term depression in hippocampal area CA1. J. Neurophysiol..

[17] Izumi Y., Zorumski C.F. (2012). NMDA receptors, mGluR5, and endocannabinoids are involved in a cascade leading to hippocampal long-term depression. Neuropsychopharmacology.

[18] Volk L.J., Daly C.A., Huber K.M. (2006). Differential roles for group 1 mGluR subtypes in induction and expression of chemically induced hippocampal long-term depression. J. Neurophysiol..

[19] Kirschstein T., Bauer M., Müller L., Rüschenschmidt C., Reitze M., Becker A.J., Schoch S., Beck H. (2007). Loss of metabotropic glutamate receptor-dependent long-term depression via downregulation of mGluR5 after status epilepticus. J. Neurosci..

[20] Johnson M.P., Kelly G., Chamberlain M. (2001). Changes in rat serum corticosterone after treatment with metabotropic glutamate receptor agonists or antagonists. J. Neuroendocrinol..

[21] Doherty A.J., Palmer M.J., Henley J.M., Collingridge G.L., Jane D.E. (1997). (RS)-2-chloro-5-hydroxyphenylglycine (CHPG) activates mGlu5, but no mGlu1, receptors expressed in CHO cells and potentiates NMDA responses in the hippocampus. Neuropharmacology.

[22] Ito I., Kohda A., Tanabe S., Hirose E., Hayashi N., Mitsunaga S., Sugiyama H. (1992). 3,5-Dihydrophenylglycine: A potent agonist of metabotropic glutamate receptors. NeuroReport.

[23] Schoepp D.D., Goldsworthy J., Johnson B.G., Salhoff C.R., Baker S.R. (1994). 3,5-Dihydroxyphenylglycine is a highly selective agonist for phosphoinositide-linked metabotropic glutamate receptors in the rat hippocampus. J. Neurochem..

[24] Ireland D.R., Abraham W.C. (2009). Mechanisms of group I mGluR-dependent long-term depression of NMDA receptor-mediated transmission at Schaffer collateral-CA1 synapses. J. Neurophysiol..

[25] Lee S.A., Oh B.M., Kim S.J., Paik N.J. (2014). The molecular evidence of neural plasticity induced by cerebellar repetitive transcranial magnetic stimulation in the rat brain: A preliminary report. Neurosci. Lett..

[26] Hu Y., Zhu Y., Wen X., Zeng F., Feng Y., Xu Z., Xu F., Wang J. (2022). Repetitive transcranial magnetic stimulation regulates neuroinflammation, relieves hyperalgesia and reverses despair-like behaviour in chronic constriction injury rats. Eur. J. Neurosci..

[27] Huang C.-C., Hsu K.-S. (2012). Activation of NMDA receptors reduces metabotropic glutamate receptor-induced long-term depression in the nucleus accumbens via a CaMKII-dependent mechanism. Neuropharmacology.

